# First-Line Treatments for Metastatic Clear Cell Renal Cell Carcinoma: An Ever-Enlarging Landscape

**DOI:** 10.1093/oncolo/oyab056

**Published:** 2022-02-01

**Authors:** Shuchi Gulati, Chris Labaki, Georgia Sofia Karachaliou, Toni K Choueiri, Tian Zhang

**Affiliations:** 1 Division of Hematology and Oncology, Department of Medicine, University of Cincinnati, Cincinnati, OH, USA; 2 Department of Medical Oncology, Dana-Farber Cancer Institute, Boston, MA, USA; 3 Division of Medical Oncology, Department of Medicine, Duke University, Durham, NC, USA; 4 Duke Cancer Institute Center for Prostate and Urologic Cancers, Durham, NC, USA; 5 Division of Hematology and Oncology, Department of Internal Medicine, UT Southwestern Medical Center, Dallas, TX, USA

**Keywords:** renal cell carcinoma, metastatic RCC, immunotherapy, new therapeutic targets

## Abstract

Treatment paradigm for metastatic clear cell renal cell carcinoma (mccRCC) has changed dramatically over the recent decades. From cytokines, interleukin-2 and interferon-α to tyrosine kinase inhibitors and mammalian target of rapamycin inhibitors, during the last decade, combinations of immune checkpoint inhibitors have taken over first-line treatment of mccRCC. These combinations are approved based on results from large phase III clinical trials, all of which used sunitinib as the comparator. These trials include CheckMate214 (ipilimumab plus nivolumab), KEYNOTE 426 (pembrolizumab plus axitinib), JAVELIN Renal 101 (avelumab plus axitinib), CheckMate 9ER (nivolumab plus cabozantinib), and the CLEAR study (lenvatinib and pembrolizumab). Results from these studies constitute milestones for newer therapeutic approaches in mccRCC. The broadening spectrum of treatment options for patients with mccRCC with multiple first-line options currently available also means that treating physicians will need to consider each option carefully, balance clinical factors, financial considerations, and weigh toxicity profiles of each drug before deciding the optimal treatment regimen for each individual patient. We describe each frontline treatment option in detail through this review to aid the decision-making process.

## Teaser

Management of metastatic clear cell renal cell carcinoma (mccRCC) has progressed to the point that immune checkpoint inhibitor (ICI) combinations or ICI/TKI combinations have become standard of care. This article highlights the current therapeutic landscape for patients with mccRCC in the first-line setting and explores the specific considerations relative to currently approved regimens, along with future perspectives.

**Implications for Practice**

This article describes the current first-line therapeutic options for patients with metastatic renal cell carcinoma (RCC) as well as the available second-line treatments for these patients. Given the recent scientific advances in elucidating further the biology of kidney cancer, the emerging agents that are being evaluated in clinical trials, as well as the potential role of the cytoreductive nephrectomy in patients with synchronous metastatic disease, are also reviewed. This article summarizes all the current changes in clinical practice regarding the RCC along with the novel agents that are currently under evaluation.

## Introduction

As of 2021, renal cell carcinoma (RCC) is among the 10 most common cancers in both men (sixth) and women (ninth) in the US.^1^ The incidence of RCC has doubled since 1975, accounting for 73 750 new cases and 14 830 deaths in 2020 in the US alone, and for approximately 2% of cancer diagnoses and deaths worldwide.^2,3^ The 5-year relative survival rate for patients with RCC was 75.6% between 2011 and 2017, with variations depending on disease stage at the time of diagnosis.^4^

High-dose interleukin (IL)-2 was the first approved immunotherapeutic agent for patients with metastatic RCC (mRCC) in 1992.^5^ Despite showing considerable efficacy and achieving a complete response (CR) rate of 5%-9%, IL-2 use has fallen out of favor due to serious adverse events (AEs) affecting multiple organ systems, and a considerable fatality rate that reached 4% in some series.^6^ High-dose IL-2 can be an option in the front-line setting; however, it should be considered only in highly selected patients with excellent performance status and requires to be administered at high-volume centers with experience in its use.^7^

Since the advent of tyrosine kinase inhibitors (TKIs) such as sorafenib and sunitinib as well as mechanistic target of rapamycin (mTOR) inhibitors such as everolimus and temsirolimus in the early 2000s, management of metastatic clear cell RCC (mccRCC) has now progressed into an era where immune checkpoint inhibitor (ICI) combinations or ICI/TKI combinations have become standard of care. Here, we highlight the current therapeutic landscape for patients with mccRCC in the first-line setting and explore the specific considerations relative to currently approved regimens, along with future perspectives.

## Histological Classification and Molecular Characterization of ccRCC

The 2016 WHO classification of kidney tumors is based on the combination of various morphological, molecular, and genetic features.^8^ From a general perspective, kidney cancer is divided into several different histological types: ccRCC represents the most common subtype (approximately 75%), followed by papillary (further divided into types 1 and 2; 15%-20%) and chromophobe histologies (approximately 5%).^9^ Other rarer subtypes include translocation RCC, renal medullary carcinoma, collecting duct carcinoma, succinate dehydrogenase-deficient RCC, and unclassified RCC. In the vast majority of ccRCCs, loss of heterozygosity at chromosome 3p (between 3p25 and 3p21 segments) can be detected. This results in the loss of tumor-suppressor genes, the most common being von Hippel-Lindau (*VHL*), followed by Polybromo 1 (*PBRM1*), BRCA1-associated protein-1 (*BAP1*), and SET domain-containing protein 2 (*SETD2*).^10-12^ A comprehensive genomic study by The Cancer Genome Atlas research network of a kidney cancer cohort included 843 patients consisting of 488 ccRCCs, 274 papillary RCCs (*n* = 160 and *n* = 70 for type 1 and type 2, respectively), and 81 chromophobe RCCs.^9^ In ccRCC, a low expression level of AMPK and a high level of ribose sugar metabolism were associated with lower survival. *PTEN* and *TP53* mutations were not associated with decreased survival rate when looking at the entire cohort; however, histological-specific identification of mutations of *PTEN* or *TP53* in RCC subtypes was associated with poor survival. *BAP1* mutation was significantly associated with decreased survival rate in the entire cohort. Additionally, T helper 2 (Th2) gene signature, DNA hypermethylation, and *CDKN2A* alterations were associated with poor prognosis among all RCC subtypes.^9^

## The International Metastatic Renal Cell Carcinoma Database Consortium Prognostic Model

Although the Memorial Sloan-Kettering Cancer Center prognostic model was first developed,^13^ the International Metastatic Renal Cell Carcinoma Database Consortium (IMDC) model is the preferred prognostic model for patients with mRCC since the development of anti-angiogenic treatments.^14^ In particular, patients with mRCC can be categorized as favorable-, intermediate-, or poor-risk disease based on clinical and laboratory risk factors.^15^ Independent risk factors for poor prognosis included in this score include a low Karnofsky performance status (cutoff: 80%), time from initial diagnosis (including localized RCC) to start of systemic therapy (ie, less than 1 year), low hemoglobin (less than the lower limit of normal [LLN]), a high neutrophil count (greater than the upper limit of normal [ULN]), hypercalcemia (corrected calcium > ULN), and thrombocytosis (platelet count> ULN).^16^ Definitions for ULN and LLN values for calculation of the IMDC score are based on local laboratory values where the test is performed, as these numbers can vary between different laboratories. Each criterion listed here is given one point and a patient with none of these criteria is considered to have a favorable-risk disease, those with 1-2 factors are considered to belong to the intermediate-risk category, and those with 3 or more criteria are considered to have a poor-risk disease. The accuracy of the IMDC model was tested and externally validated, and it has since been used to stratify patients for randomized treatments in contemporary clinical trials, including ICI trials.^16^

## Systemic Treatment Approach of Patients with mRCC in First-line Treatment

During the last decade, the landscape of first-line therapeutic regimens for patients with mccRCC has widely expanded. The first US Food and Drug Administration (FDA) approved ICI agent in patients with mRCC was nivolumab, a programmed death protein-1 (PD-1) inhibitor. This approval was based on the CheckMate 025 phase III clinical trial (NCT01668784) in 2015, where the novel agent was compared with everolimus (mTOR inhibitor), showing substantial benefit in terms of overall survival (OS) with a hazard ratio (HR) for risk of death (any cause) with nivolumab versus everolimus of 0.73 (98.5% confidence interval [CI], 0.57 to 0.93).^17^ Subsequently, 6 randomized clinical trials (RCTs) have investigated the safety and efficacy of immunotherapeutic agents (PD-1 and programmed cell death ligand 1 [PD-L1] inhibitors) in combination with cytotoxic T-lymphocyte-associated protein-4 (CTLA-4) inhibitor ipilimumab or vascular endothelial growth factor (VEGF) TKIs, where the comparator cohort was treated with sunitinib (Table 1). Only one study (CABOSUN), a randomized phase II trial evaluated a single agent TKI (cabozantinib) arm in comparison to sunitinib. This study showed progression-free survival (PFS) and objective response rate (ORR) benefits but was not powered for OS.^18^ In the subsequent sections, we describe these combination clinical trials which represent the main focus of first-line therapeutic approaches in mccRCC

**Table 1.** Summary of the completed phase III clinical trials for first-line treatment of patients with metastatic RCC.

ClinicalTrials.gov ID	Number of participants	Interventional agent	Median OS Months HR (CI)	Median PFS Months HR (CI)
NCT02811861 (CLEAR study)	1069	Lenvatinib + pembrolizumab OR Lenvatinib/everolimus vs sunitinib	NR vs NR vs NR 0.66 (0.49 to 0.88), 1.15 (0.88 to 1.50)	23.9 vs 15.0 vs 9.2 0.39 (0.32 to 0.49), 0.65 (0.53 to 0.80)
NCT02231749 (CheckMate 214 study)	1096	Nivolumab + ipilimumab vs sunitinib	55.7 vs 38.4 0.72 (0.62 to 0.85)	12.3 vs 12.3 0.86 (0.73 to 1.01)
NCT03141177 (CheckMate 9ER study)	651	Nivolumab + cabozantinib vs sunitinb	NR vs 29.5 0.66 (0.50 to 0.87)	17.0 vs 8.3 0.52 (0.43 to 0.64)
NCT02684006 (JAVELIN Renal 101study)	886	Avelumab + axitinib vs sunitinib	NR vs NR 0.79 (0.62 to 1.03)	13.3 vs 8.0 0.69 (0.57 to 0.83)
NCT02853331 (KEYNOTE 426 study)	861	Pembrolizumab+ axitinib vs sunitinib	45.7 vs 40.1 0.73 (0.60 to 0.88)	15.7 vs 11.1 0.68 (0.58 to 0.80)
NCT02420821 (IMmotion 151)	454	Bevacizumab + atezolizumab vs sunitinib	33.6 vs 34.9 0.93 (0.76 to 1.14)	11.2 vs 8.4 0.83 (0.70 to 0.97)

CI, confidence interval; HR, hazard ratio, NR, not reached; OS, overall survival; PFS, progression-free survival; RCC, renal cell carcinoma.

CheckMate 214 (NCT02231749), a phase III RCT, assigned 1096 patients to either nivolumab plus ipilimumab (a CTLA-4 inhibitor; *n* = 550), or sunitinib (*n* = 546).^19^ The combination demonstrated an improved OS in patients with intermediate- and poor-risk (IMDC) disease, with an HR of 0.63 (99.8% CI, 0.44 to 0.89), based on which it received FDA approval in 2018. In a subsequent updated analysis at a median follow-up of 55.0 months, OS remained superior for the concurrent administration of nivolumab plus ipilimumab (median OS: not reached vs 38.4 months, respectively; HR 0.65; 95% CI, 0.59 to 0.81).^20^ Patients with an IMDC intermediate/poor risk achieved a median OS of 48.1 with the combination versus 26.6 months with sunitinib (HR 0.65; 95% CI, 0.54 to 0.78). Sunitinib continues to perform better for the IMDC good-risk group (HR for PFS: 1.84; 95% CI, 1.29 to 2.62), although this difference was less prominent than in earlier analyses. Importantly, durable responses were achieved with approximately 30% of patients without disease progression at 5-year follow-up. Additionally, CRs were seen in 59 (10.7%) patients who received nivolumab/ipilimumab versus 14 patients (2.6%) who received sunitinib. Of the 59 patients who achieved CR with nivolumab/ipilimumab; 27 patients (45.8%) had stopped therapy and had not required subsequent systemic therapy. Treatment-free survival intervals are also important; when compared with patients who received sunitinib, patients who received ipilimumab-nivolumab had more breaks off of systemic therapy and more time without side effects.^21^

The combination of pembrolizumab and axitinib (a VEGFR-targeted TKI) was evaluated in the phase III KEYNOTE 426 clinical trial, using sunitinib as a comparator.^22^ Eight hundred sixty-one patients with previously untreated advanced RCC were randomly assigned to receive pembrolizumab plus axitinib (*n* = 432) or sunitinib (*n* = 429). At a median follow-up of 42.8 months in the latest updated analysis, the combination of pembrolizumab plus axitinib demonstrated a benefit for OS when compared with sunitinib (median OS: 45.7 vs 40.1 months, respectively; HR 0.73, 95% CI, 0.60 to 0.88). Median PFS in the combination cohort was 15.7 months compared with 11.1 months for sunitinib (HR 0.68, 95% CI, 0.58 to 0.80). Long-term follow-up also shows a promising response rate with an ORR for pembrolizumab plus axitinib of 60.4% versus 39.6% for sunitinib (*P* < .0001); and a CR rate of 10.0% versus 3.5%.^23^

Importantly, the association of pembrolizumab plus axitinib showed a clinical benefit in all subgroups (based on the IMDC risk score as well as on PD-L1 expression). Based on these results, it received FDA approval in 2019.^24^

IMmotion151 (NCT02420821) was a phase III RCT comparing the combination of atezolizumab (a PD-L1 inhibitor) plus bevacizumab (anti-VEGF; *n* = 454) to sunitinib (*n* = 461).^25^ The co-primary endpoint of PFS in the PD-L1-positive population was met, with a median of 11.2 months in the combination arm as opposed to 7.7 months with sunitinib (HR 0.74, 95% CI, 0.57 to 0.96; *P* = .02). OS in the intention to treat (ITT) population (co-primary endpoint) did not cross the prespecified significance boundary (stratified HR 0.84, 95% CI, 0.62 to 1.15; *P* = .29) and consequently, the combination did not receive regulatory approval. Integrated multi-omics analyses using RNA transcriptomics from 823 tumor samples in an unsupervised manner in the IMmotion151 trial revealed 7 molecular subgroups.^26^ The subgroups included the highly angiogenic clusters 1 and 2 (enriched for vascular and VEGF pathway-related genes), with cluster 1 being more enriched for stroma-specific expression. Cluster 3 was characterized by the expression of cell cycle genes and genes associated with the complement cascade. Clusters 4, 5, and 6 were enriched for cell cycle transcriptional signatures as well as anabolic metabolism-related genes (FAS and pentose phosphate pathways). In addition, cluster 4 exhibited a high expression of T-effector, JAK/STAT, and interferon a/ɣ gene expression profiles while clusters 5/6 were enriched for the previously described myeloid Inflammation gene signature.^26^ Cluster 7 showed a high expression of snoRNAs, which have been implicated in epigenetic changes linked to carcinogens. Overall, this analysis provides a preliminary molecular basis to define patient subgroups as well as response to treatment regimens and needs to be further validated in RCC well as other tumor types where IO-based regimens are being used. Although these classifiers are promising for tumor selection, a trial prospectively utilizing these gene expression clusters to designate individualized therapy would be required to validate their role as a biomarker in RCC. A potential example would be a trial where tumors enriched for clusters 1 and 2 could be assigned to receive an ICI with a VEGF-TKI, whereas clusters 4, 5, and 7 may not require a TKI. Such a prospective trial is eagerly awaited.

Another frontline phase III study, JAVELIN Renal 101, compared avelumab (PD-L1 inhibitor) plus axitinib (*n* = 442) to sunitinib (*n* = 444).^27^ With a minimum follow-up of 13 months in all patients, in the updated analysis, PFS in the overall population was 13.3 months in the avelumab and axitinib combination arm versus 8.0 months in the sunitinib arm (HR 0.69, 95% CI, 0.57 to 0.82).^27^ Of note, the OS is not mature yet with a median duration of follow-up of 19.3 months in the combination arm and 19.2 months in the sunitinib arm. The ORR was 52.5% in the combination arm with a CR rate of 3.8% arm as opposed to 27.3% and 2%, respectively, in the sunitinib arm. In light of these results, the combination received FDA approval in 2019. Importantly, subsequent biomarker analysis from JAVELIN Renal 101 showed that PD-L1 expression and tumor mutational burden were not predictive of PFS in either cohort. The JAVELIN Renal 101 Immuno-signature, composed of 26 genes related to T cells, NK cells, and chemokines, helped to identify responders (vs nonresponders) in the avelumab plus axitinib group and was further validated in an independent dataset (the phase Ib JAVELIN Renal 100 trial). Similarly, a 26-gene signature helping to differentiate PFS in patients treated with sunitinib was discovered (named the JAVELIN Angio signature). Interestingly, the differentiating ability of the JAVELIN Immuno and Angio signatures were specific for the combination of avelumab plus axitinib, and sunitinib, respectively.^27,28^ In terms of clinical practice and according to a recently published analysis across 11 international kidney cancer centers, the combination of axitinib plus avelumab is not the preferred choice among clinicians due to the lack of OS data.^29^

Another IO/TKI combination to receive FDA approval in January 2021 is the combination of nivolumab plus cabozantinib,^30,31^ based on the data from the CheckMate 9ER study (NCT03141177) in patients with previously untreated mRCC. CheckMate 9ER is an open-label, multi-national phase III RCT that investigated the combination of nivolumab plus cabozantinib (40 mg/day) compared with sunitinib.^30,32^ Preclinical rationale to combine the drugs comes from data regarding nivolumab’s ability to prevent cancer from evading immune detection^33,34^ and cabozantinib’s immunomodulatory and antiangiogenic properties derived from the inhibition of multiple tyrosine kinases (including VEGF-R, MET, and AXL).^35,36^ With a median follow-up of 18.1 months, median PFS was 16.6 months (95% CI, 12.5 to 24.9) in the nivolumab and cabozantinib arm versus 8.3 months (95% CI, 7.0 to 9.7) in the sunitinib arm; HRs for progressive disease and death were 0.51 (95% CI, 0.41 to 0.64) and 0.60 (95% CI, 0.40 to 0.89), respectively. The combination of nivolumab and cabozantinib displayed an ORR of 55.7%, when compared with 27.1% with sunitinib.^30^

The latest clinical trial that reported the efficacy of combining targeted agents with ICIs was the CLEAR study^37^ or Keynote 581 (NCT02811861).^38^ This was a multicenter, randomized, open-label phase III trial, aiming to evaluate the combination of lenvatinib with prembrolizumab in patients with mRCC. Of note, CLEAR differs from the previous landmark trials in that it included 3 treatment arms: pembrolizumab plus lenvatinib (1), lenvatinib plus everolimus (2), and sunitinib as control (3). One thousand sixty-nine patients were randomized to receive lenvatinib plus everolimus (*n* = 357), pembrolizumab plus lenvatinib (*n* = 355), or sunitinib (*n* = 357). At a median follow-up of 26.6 months, both combinations of lenvatinib plus pembrolizumab (median PFS 23.9 months; HR 0.39; 95% CI, 0.32 to 0.49) and lenvatinib plus everolimus (median PFS 14.7 months; HR 0.65; 95% CI, 0.53 to 0.80) displayed lower progression rates compared with sunitinib (median PFS 9.2 months).^37^ While median OS was not reached in any of the treatment cohorts, a survival benefit was seen for the lenvatinib plus pembrolizumab group compared with sunitinib (HR for death 0.66; 95% CI, 0.49 to 0.88; *P* = .005). The ORR for patients treated with pembrolizumab plus lenvatinib was 71% (with 16% patients achieving a CR), compared with 54% (CR: 10%) for patients receiving lenvatinib plus everolimus and 36% (CR: 4%) in the sunitinib arm.

An important point to note in this study was the dose of lenvatinib (20 mg/day) used in combination arm with pembrolizumab, higher than the 18 mg/day dose used in combination with everolimus and which is the current standard in clinical practice. This dosing regimen may have contributed to 82.4% of patients experiencing grade 3 or higher AEs in the lenvatinib plus pembrolizumab cohort. However, even in the lenvatinib/everolimus arm, grade 3/4 AEs were seen in 83.1% of patients, compared with 71.8% with sunitinib. To summarize, the CLEAR study adds another IO/targeted therapy combination to the armamentarium of available options for the treatment of patients with advanced RCC in the frontline setting, and is expected to receive regulatory approval in the near future.

As several treatment options become available for patients with mccRCC, clinicians will be faced with the conundrum of how to choose the ideal treatment and how to sequence these treatments regimens. Novel clinical trial designs based on patient selection or treatment sequencing will be required to answer these critical issues. While efficacy is important, the effect of each treatment on quality of life (QoL) should also be taken into consideration when deciding which regimen to use in the first-line setting. In both CheckMate 9ER^39^ and KEYNOTE 426^40^ studies, the rate of treatment discontinuation related to AEs was reported to be low (at 5.6% for cabozantinib plus nivolumab^39^ and 7% for pembrolizumab plus axitinib).^40^ Furthermore, in CheckMate 214^20^ and CheckMate 9ER^39^ studies, an improvement in global patient-reported outcomes (PROs) (using the Functional Assessment of Cancer Therapy–Kidney Cancer Symptom Index-19 and EQ-5D-3L instruments) were reported with both nivolumab and ipilimumab as well as with nivolumab and cabozantinib, over sunitinib, respectively. KEYNOTE-426^40^ and CLEAR^37^ reported no major decline in PROs when treated with pembrolizumab/ axitinib^40^ and lenvatinib/pembrolizumab, respectively.^37^ It is important to remember that even if all the studies had sunitinib as control arm at 50 mg/day (4 days-on, 2 days-off schedule), the PROs types and schedules of immunotherapeutic combinations were different among studies, preventing any cross-trial comparison for this metric.

**Figure 1.**
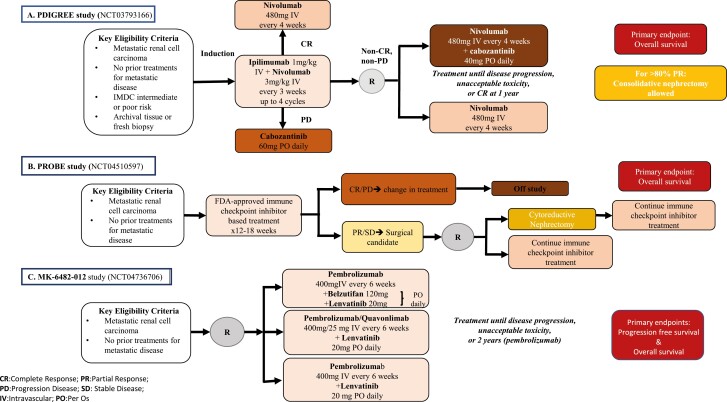
Phase III clinical trials actively accruing patients with metastatic RCC.

Furthermore, the data from ongoing trials will help elucidate sequencing and combination approaches. The PDIGREE study (NCT03793166), a randomized, multicenter phase III trial^41^ aims to answer immunotherapy sequencing questions. This study allows patients with IMDC intermediate or poor-risk mccRCC to start the combination of nivolumab plus ipilimumab as induction for up to 4 cycles (Fig. 1). Further treatment (ie, maintenance nivolumab, cabozantinib monotherapy, or the combination of both drugs) is then adapted based on initial response as measured radiographically at 3 months. While the primary endpoint is the OS assessed at up to 5 years, the CR rate, PFS, and ORR are key secondary endpoints. Importantly, this is the first trial in mRCC to prospectively discontinue treatment for complete responders at 1 year. Additionally, PDIGREE does allow for consolidative nephrectomy for those patients who achieve excellent partial responses. The study is enrolling well across the US.

**Figure 2.**
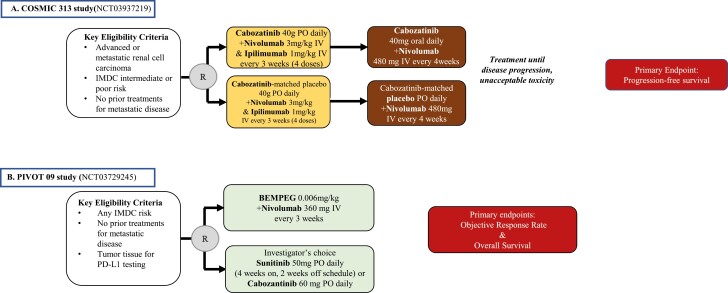
First-line phase III clinical trials with completed enrollment for patients with metastatic RCC, awaiting data.

The role of triple therapy in the management of advanced RCC is also being evaluated in 2 separate trials. The COSMIC 313 study (NCT03937219)^42^ is a global, randomized, placebo-controlled, double-blind, phase III trial that further builds on the efficacy of the combination of nivolumab plus cabozantinib in patients with intermediate or poor-risk disease per IMDC criteria (Fig. 2). With a planned enrollment of 840 patients, this trial investigates the efficacy and safety of the concurrent administration of cabozantinib, nivolumab, and ipilimumab compared with nivolumab plus ipilimumab. The primary endpoint is PFS, and secondary key endpoints include OS, ORR, duration of response, safety, and correlation of biomarkers with clinical outcomes. A second trial is also evaluating the potential benefit of triplets with the addition of belzutifan or quavonlimab (a CTLA-4 inhibitor) to lenvatinib and pembrolizumab (NCT04736706). The results from these studies will show the potential benefit versus toxicities of upfront triplet treatments.

Finally, the PIVOT 09 study (NCT03729245) that recently finished accrual is evaluating bempegaldesleukin (BEMPEG), first-in-class pegylated IL-2 receptor agonist, with nivolumab, compared with the investigator’s choice of anti-VEGF TKI, in treatment-naïve patients. This trial is based on the results of a phase I/II study that investigated the efficacy and safety of the combination of BEMPEG and nivolumab, and showed an encouraging ORR in advanced RCC in addition to an acceptable safety profile.^43^ Primary endpoints include ORR and OS, both in the overall population and IMDC intermediate- and poor-risk patients. Secondary endpoints include OS in the PD-L1-positive population, PFS, safety, and QoL.^44^

## Sequencing in mRCC: What to Do in Second Line?

Despite the tremendous progress in frontline therapeutic options in kidney cancer, there are still many patients who need to be treated with second-line and beyond treatments. Agents studied in these settings are briefly described here.

In patients with advanced mccRCC who had progressed on a VEGFR TKI, the efficacy and safety of the VEGFR/MET/Axl TKI cabozantinib versus the mTOR inhibitor everolimus was investigated in the multicenter randomized phase III clinical trial, METEOR (NCT1865747).^45,46^ Six hundred fifty-eight patients were randomized to receive cabozantinib or everolimus. At a median follow-up of 18.8 months, the median OS was 21.4 months (95% CI, 18.7 to not reached) in the cabozantinib group compared with 16.5 months (95% CI, 14.7 to 18.8) with everolimus groups (HR 0.66; 95% CI, 0.53 to 0.83). Improvements in PFS (HR 0.51; 95% CI, 0.41 to 0.62) and ORR (17% with cabozantinib vs 3% with everolimus; *P* < .0001) were also seen. Grades 3-4 AEs were reported in 71% versus 60% patients treated with cabozantinib and everolimus, respectively, making cabozantinib administration at this dose slightly challenging, similar to what has been seen in first-line combination therapies in the first-line setting.

Lenvatinib was evaluated in a randomized-controlled multicenter phase II trial (NCT01136733), alone or in combination with everolimus in patients with advanced RCC who had progressed on VEGF TKI treatment.^47^ Patients were randomized 1:1:1 to receive lenvatinib versus everolimus versus the combination of the 2 drugs. The combination of lenvatinib plus everolimus significantly prolonged PFS when compared with everolimus alone (median PFS of 14.6 vs 5.5 months; HR 0.40; 95% CI, 0.24 to 0.68), but not compared with lenvatinib alone (median PFS of 7.4 months; HR 0.66; 95% CI, 0.39 to 1.10; *P* = .12). Lenvatinib monotherapy also significantly prolonged PFS compared with everolimus (HR 0.61; 95% CI, 0.38 to 0.98). Grades 3-4 AEs were seen in 50% of patients treated with single-agent everolimus, compared with 79% of patients with lenvatinib monotherapy and 71% with both drugs.^47^ Based on these results, the combination of lenvatinib and everolimus received FDA approval in 2016.

Another open-label, randomized, controlled, phase III clinical trial, TIVO-3 (NCT02627963) evaluated the efficacy and safety of tivozanib as third or fourth-line treatment in patients with mRCC compared with those who were treated with sorafenib.^48^ At a median follow-up of 19.0 months, the PFS was significantly longer in tivozanib group over sorafenib (5.6 vs 3.9 months, respectively; HR 0.73; *P* = .02) and the ORR was superior for tivozanib compared with sunitinib (18 vs 8%, respectively; *P* = .02).^49^ Serious treatment-related AEs were reported in 19 (11%) patients treated with tivozanib and in 17 (10%) patients treated with sorafenib while deaths related to treatment were not identified. Based on the results of this study, in March 2021, the US FDA approved tivozanib for adult patients with relapsed or refractory advanced mccRCC previously treated with 2 or more systemic therapies.^50^

A promising combination currently being investigated in an ongoing phase III trial is the combination of cabozantinib with atezolizumab versus cabozantinib monotherapy in patients with mRCC previously treated with immunotherapy (NCT04338269).^51^ The primary endpoints of this study are PFS and OS in the ITT, while the secondary endpoints include ORR and duration of response. TiNivo-2 is another phase III trial evaluating second-line options in patients with mccRCC previously treated with ICIs, with the combination of tivozanib and nivolumab. The study is expected to begin enrollment in mid-2021.^52^

## Novel Therapeutic Agents for mccRCC

With recent scientific advances helping to further elucidate the biology of kidney cancer, novel avenues are being explored for the development of new therapeutic options for patients with RCC. Hypoxia-inducible factor-2α (HIF-2α), a transcription factor, has been shown to accumulate in kidney cancer due to the loss of the *VHL* gene, causing the activation of downstream messengers, and has been consequently identified as a critical target in RCC. Following this major breakthrough, HIF-2α inhibitors are currently under evaluation in patients with mRCC. In phase I dose-escalation trial with advanced ccRCC,^53,54^ the first-generation HIF-2α inhibitor MK-3795 (PT2385) displayed a promising safety profile with no patients discontinuing therapy due to AEs and led to an ORR of 14% and a disease-control rate of 66% in 51 heavily pretreated patients. As highly variable pharmacokinetics were identified for PT2385, a second generation of HIF-2α inhibitors, belzutifan (MK-6482, previously known as PT2977) has been developed, achieving a higher potency and a greater selectivity. Belzutifan was assessed in an open-label phase I trial (NCT02974738) in patients with advanced ccRCC with at least one prior treatment.^55-57^ Among the 55 patients enrolled in this trial, the median PFS was 14.5 months, and the ORR was 25% with a disease control rate of 80%. Of note, the activity of belzutifan was noted across all IMDC risk groups. Belzutifan is currently being assessed in multiple trials as a monotherapy or in combination with other established drugs. Examples include NCT04195750, which is a multicenter, randomized phase III clinical trial evaluating the efficacy of belzutifan compared with everolimus in patients with previously treated advanced ccRCC.^58^ NCT03634540 is a phase II open-label trial, where the combination of belzutifan plus cabozantinib is being evaluated for patients with advanced ccRCC in treatment naïve patients (cohort 1) or those previously treated with ICIs (cohort 2). Preliminary results for cohort 2 at a median follow-up of 11.3 months^59^ showed an ORR of 22.0% and a disease control rate of 92.7%. The median PFS was 16.8 months (95% CI, 9.2-not reached) and the OS rate at 6 months reached 95%. These promising results outline the efficacy of HIF-2α inhibitors in heavily pre-treated populations with ccRCC, and more evidence is awaited regarding treatment-naïve patients.

Anaplerotic reprogramming along with an altered glucose metabolism have been identified as hallmarks of RCC. This is exemplified with the entry of glutamine to the tricarboxylic acid cycle (TCA) cycle through ketoglutarate (called glutamine-derived ketoglutarate), enabling the replenishment of TCA intermediates that have been used for biosynthesis.^60^ Based on this preclinical evidence, a glutaminase inhibitor, telaglenastat (CB-839) was evaluated in phase I and phase II trials when combined with everolimus (NCT03163667)^61^ or cabozantinib (NCT02071862, NCT03428217^62^) in patients with heavily pre-treated mRCC.^63^ Subsequently, results from one of these trials (ENTRATA), where the drug is combined with everolimus showed an improved PFS of 3.8 months compared with 1.9 months in the placebo plus everolimus group (HR 0.64, one-sided *P* = .079 [threshold for significance: 0.2]).^64^ Although this was a promising agent in early trials, the CANTATA team recently presented that the combination of telaglenastat and cabozantinib compared with cabozantinib failed to meet its primary endpoint, with a median PFS of 9.2 months versus 9.3 months respectively (HR 0.94, *P* = .65).^65^

## Cytoreductive Nephrectomy: A Potential Role in Synchronous Metastatic Disease

The role of upfront cytoreductive nephrectomy (CN) has been investigated in recent years in 2 large studies. In the SURTIME study, no difference in PFS was identified between the deferred versus immediate CN cohorts (HR 0.88; 95% CI, 0.56 to 1.37; *P* = .57^66^), whereas the OS did differ with a median OS of 32.4 months in the deferred CN group compared with 15.0 months for immediate CN (HR 0.57; 95% CI, 0.34 to 0.95).^66^ In the updated analysis of the CARMENA trial,^67^ which compared sunitinib alone versus after nephrectomy in patients with mRCC, no difference in OS was identified in the ITT population at a follow-up of 61.5 months (median OS of 23.6 vs 22.7 months, respectively; HR 1.08; 95% CI, 0.75 to 1.57). However, subgroup analysis showed a large heterogeneity between the different categories of patients. In fact, while CN was associated with a worse survival in patients with 2 IMDC risk factors when compared with those receiving sunitinib only (median OS: 16.6 vs 31.2 months, respectively; HR 0.61; 95% CI, 0.41 to 0.91), a potential benefit was identified in those with only one IMDC risk factor (median OS: 30.5 vs 25.2 months, respectively; HR 1.24; 95% CI, 0.81 to 1.90). Furthermore, in a propensity-score-based analysis from the IMDC group CN was associated with a significantly better OS in patients receiving targeted therapies (HR 0.56; 95% CI, 0.51 to 0.62) and ICIs (HR 0.39; 95% CI, 0.19 to 0.83).^68^ Another important aspect of CARMENA is that this was a noninferiority trial and such trials have been shown to produce desirable results (ie, noninferiority for the experimental treatment) in greater than 80% of the studies.^69,70^ Regardless, CN remains an important component in the therapeutic algorithm of patients with advanced kidney cancer. However, this approach remains to be better tailored, as only selected subgroups of patients might benefit from it.

The ongoing research regarding the optimal timing of immunotherapy in relation to CN in patients with mRCC includes 4 therapeutic clinical trials in the US National Clinical Trials Network (Table 2). The PROBE study (NCT04510597), activated in November 2020, randomizes patients with mRCC to an immunotherapy-based combination, with or without consolidative nephrectomy. The primary endpoint of PROBE is OS. Another phase III trial, NORDIC-SUN (NCT03977571), investigates the role of CN in patients with mRCC receiving ipilimumab and nivolumab. Patients with fewer than 3 IMDC risk features and a resectable tumor are randomized after 4 cycles of combination nivolumab and ipilimumab to maintenance nivolumab with or without CN. The primary endpoint of NORDIC-SUN is OS, while secondary endpoints include PFS and ORR. In addition, a phase II clinical trial (CYTOSHRINK-NCT04090710)^71^ also investigates the efficacy of the upfront cytoreductive stereotactic body radiation therapy (SBRT) in combination with the standard of care nivolumab plus ipilimumab over the combination of nivolumab plus ipilimumab alone in patients with advanced RCC and IMDC intermediate/poor-risk disease, who were not suitable for CN or have declined it. The primary endpoint of this study is the PFS while OS, ORR, and HRQoL are included as secondary objectives.

**Table 2.** Summary of the ongoing clinical trials for first-line metastatic RCC.

ClinicalTrials.gov ID	Interventions	Phase (planned number of participants)
NCT04510597 (PROBE study)	Immunotherapy-based combination with cytoreductive nephrectomy vs no cytoreductive nephrectomy	3 (*n* = 364)
NCT03793166 (PDIGREE study)	Nivolumab + ipilimumab followed by randomization to nivolumab vs nivolumab+cabozantinib	3 (*n* = 1046)
NCT04736706 (MK-6482-012 study)	Pembrolizumab + belzutifan + lenvatinib vs pembrolizumab + quavonlimab + lenvatinib vs pembrolizumab+lenvatinib	3 (*n* = 1431)
NCT03937219 (COSMIC 313)	Cabozantinib + nivolumab + ipilimumab vs nivolumab + ipilimumab + placebo	3 (*n* = 676)
NCT03729245 (PIVOT 09 study)	Bempegaldesleukin + nivolumab vs sunitinib or cabozantinib	3 (*n* = 600)

RCC, renal cell carcinoma.

The results from these studies along with those from the PDIGREE^41^ trial that allows consolidative nephrectomy for patients who achieve excellent partial responses will inform patient selection for future clinical decision-making on timing and applicability of CN.

## Conclusions

The spectrum of the available treatments for kidney cancer in the metastatic setting has widely expanded during the last decade. Upon the approval of more combination therapies, immunotherapy agents constitute the backbone for both first- and second-line treatments. The recent approvals of both combinations nivolumab plus cabozantinib and pembrolizumab plus lenvatinib add further treatment options to the frontline therapeutic armamentarium for patients with mccRCC. Undoubtedly, better CR rates are more frequently achieved, leading to longer durations of response with combination regimens. When having first-line treatment discussions for mccRCC, clinicians should be advised to present options that align with patients’ presentations and goals—balancing the goals for CRs, early disease control, potential need for sequential treatments, and risks for upfront versus long-term toxicities in the context of individual patient comorbidities. There are still many unmet medical needs in the field of RCC at the clinical and translational science levels, including among others rare variants of kidney cancers.^72^ The completion of ongoing randomized clinical trials will further shed light on more effective and tolerable systemic treatments, addressing some of the existing questions regarding optimal treatment sequencing as well as timing of both surgical and radiation treatments. Further efforts should be invested in future studies to identify patient characteristics and establish biomarkers to tailor optimal, personalized therapeutic algorithms for patients with mccRCC.

## Data Availability

The data underlying this article will be shared at reasonable request to the corresponding author.

## References

[1] Siegel RL , Miller KD, Fuchs HE, Jemal A. Cancer statistics, 2021. CA Cancer J Clin. 2021;71(1):7-33. 33433946 10.3322/caac.21654

[2] Siegel RL , Miller KD, Jemal A. Cancer statistics, 2020. CA Cancer J Clin. 2020;70(1):7-30. 31912902 10.3322/caac.21590

[3] Padala SA , Barsouk A, Thandra KC, et al Epidemiology of renal cell carcinoma. World J Oncol. 2020;11(3):79-87. 32494314 10.14740/wjon1279 PMC7239575

[4] Howlader N , Noone AM, Krapcho M, et al SEER Cancer Statistics Review, 1975-2018. Based on November 2020 SEER data submission, posted to the SEER website, April 2021. National Cancer Institute. https://seer.cancer.gov/csr/1975_2018/

[5] Fyfe G , Fisher RI, Rosenberg SA, Sznol M, Parkinson DR, Louie AC. Results of treatment of 255 patients with metastatic renal cell carcinoma who received high-dose recombinant interleukin-2 therapy. J Clin Oncol. 1995;13(3):688-696. 7884429 10.1200/JCO.1995.13.3.688

[6] Schwartz RN , Stover L, Dutcher JP. Managing toxicities of high-dose interleukin-2. Oncology (Williston Park). 2002;16(11 suppl 13):11-20. 12469935

[7] National Comprehensive Cancer Network. *NCCN Guidelines for Treatment of Cancer by Site*. National Comprehensive Cancer Network; 2019.

[8] Moch H , Cubilla AL, Humphrey PA, Reuter VE, Ulbright TM. The 2016 WHO Classification of tumours of the urinary system and male genital organs-part a: renal, penile, and testicular tumours. Eur Urol. 2016;70(1):93-105. 26935559 10.1016/j.eururo.2016.02.029

[9] Ricketts CJ , De Cubas AA, Fan H, et al; Cancer Genome Atlas Research Network. The cancer genome atlas comprehensive molecular characterization of renal cell carcinoma. Cell Rep. 2018;23(1):313-326.e5. 29617669 10.1016/j.celrep.2018.03.075 PMC6075733

[10] Cancer Genome Atlas Research Network. Comprehensive molecular characterization of clear cell renal cell carcinoma. Nature. 2013;499(7456):43-49. 23792563 10.1038/nature12222 PMC3771322

[11] Gatto F , Nookaew I, Nielsen J. Chromosome 3p loss of heterozygosity is associated with a unique metabolic network in clear cell renal carcinoma. Proc Natl Acad Sci USA. 2014;111(9):E866-E875. 24550497 10.1073/pnas.1319196111 PMC3948310

[12] Czyzyk-Krzeska MF , Landero Figueroa JA, Gulati S, et al Molecular and metabolic subtypes in sporadic and inherited clear cell renal cell carcinoma. Genes (Basel). 2021;12(3):388. 33803184 10.3390/genes12030388 PMC7999481

[13] Motzer RJ , Mazumdar M, Bacik J, Berg W, Amsterdam A, Ferrara J. Survival and prognostic stratification of 670 patients with advanced renal cell carcinoma. J Clin Oncol. 1999;17(8):2530-2540. 10561319 10.1200/JCO.1999.17.8.2530

[14] Heng DY , Xie W, Regan MM, et al Prognostic factors for overall survival in patients with metastatic renal cell carcinoma treated with vascular endothelial growth factor-targeted agents: results from a large, multicenter study. J Clin Oncol. 2009;27(34):5794-5799. 19826129 10.1200/JCO.2008.21.4809

[15] Choueiri TK , Motzer RJ. Systemic therapy for metastatic renal-cell carcinoma. N Engl J Med. 2017;376(4):354-366. 28121507 10.1056/NEJMra1601333

[16] Heng DY , Xie W, Regan MM, et al External validation and comparison with other models of the international metastatic renal-cell carcinoma database consortium prognostic model: a population-based study. Lancet Oncol. 2013;14(2):141-148. 23312463 10.1016/S1470-2045(12)70559-4 PMC4144042

[17] Motzer RJ , Escudier B, McDermott DF, et al; CheckMate 025 Investigators. Nivolumab versus everolimus in advanced renal-cell carcinoma. N Engl J Med. 2015;373(19):1803-1813. 26406148 10.1056/NEJMoa1510665 PMC5719487

[18] Choueiri TK , Hessel C, Halabi S, et al Cabozantinib versus sunitinib as initial therapy for metastatic renal cell carcinoma of intermediate or poor risk (Alliance A031203 CABOSUN randomised trial): progression-free survival by independent review and overall survival update. Eur J Cancer. 2018;94:115-125. 29550566 10.1016/j.ejca.2018.02.012 PMC6057479

[19] Motzer RJ , Rini BI, McDermott DF, et al; CheckMate 214 Investigators. Nivolumab plus ipilimumab versus sunitinib in first-line treatment for advanced renal cell carcinoma: extended follow-up of efficacy and safety results from a randomised, controlled, phase 3 trial. Lancet Oncol. 2019;20(10):1370-1385. 31427204 10.1016/S1470-2045(19)30413-9 PMC7497870

[20] Albiges L , Tannir NM, Burotto M, et al Nivolumab plus ipilimumab versus sunitinib for first-line treatment of advanced renal cell carcinoma: extended 4-year follow-up of the phase III CheckMate 214 trial. ESMO Open. 2020;5(6):e001079. 33246931 10.1136/esmoopen-2020-001079 PMC7703447

[21] Regan MM , Jegede OA, Mantia CM, et al Treatment-free survival after immune checkpoint inhibitor therapy versus targeted therapy for advanced renal cell carcinoma: 42-month results of the checkmate 214 trial. Clin Cancer Res. 2021;27(24):6687-6695. 34759043 10.1158/1078-0432.CCR-21-2283 PMC9357269

[22] Rini BI , Plimack ER, Stus V, et al; KEYNOTE-426 Investigators. Pembrolizumab plus axitinib versus sunitinib for advanced renal-cell carcinoma. N Engl J Med. 2019;380(12):1116-1127. 30779529 10.1056/NEJMoa1816714

[23] Rini BI , Plimack ER, Stus V, et al Pembrolizumab (pembro) plus axitinib (axi) versus sunitinib as first-line therapy for advanced clear cell renal cell carcinoma (ccRCC): Results from 42-month follow-up of KEYNOTE-426. J Clin Oncol. 2021;39(suppl 15; abstr 4500).

[24] U.S. Food and Drug Administration. FDA approves pembrolizumab plus axitinib for advanced renal cell carcinoma. https://www.fda.gov/drugs/drug-approvals-and-databases/fda-approves-pembrolizumab-plus-axitinib-advanced-renal-cell-carcinoma. Accessed November 2021.

[25] Rini BI , Powles T, Atkins MB, et al; IMmotion151 Study Group. Atezolizumab plus bevacizumab versus sunitinib in patients with previously untreated metastatic renal cell carcinoma (IMmotion151): a multicentre, open-label, phase 3, randomised controlled trial. Lancet. 2019;393(10189):2404-2415. 31079938 10.1016/S0140-6736(19)30723-8

[26] Motzer RJ , Banchereau R, Hamidi H, et al Molecular subsets in renal cancer determine outcome to checkpoint and angiogenesis blockade. Cancer Cell. 2020;38(6):803-817.e4. 33157048 10.1016/j.ccell.2020.10.011 PMC8436590

[27] Choueiri TK , Motzer RJ, Rini BI, et al Updated efficacy results from the JAVELIN Renal 101 trial: first-line avelumab plus axitinib versus sunitinib in patients with advanced renal cell carcinoma. Ann Oncol. 2020;31(8):1030-1039. 32339648 10.1016/j.annonc.2020.04.010 PMC8436592

[28] Motzer RJ , Robbins PB, Powles T, et al Avelumab plus axitinib versus sunitinib in advanced renal cell carcinoma: biomarker analysis of the phase 3 JAVELIN Renal 101 trial. Nat Med. 2020;26(11):1733-1741. 32895571 10.1038/s41591-020-1044-8 PMC8493486

[29] Aeppli S , Schmaus M, Eisen T, et al First-line treatment of metastatic clear cell renal cell carcinoma: a decision-making analysis among experts. ESMO Open. 2021;6(1):100030. 33460963 10.1016/j.esmoop.2020.100030 PMC7815472

[30] Cella D , Choueiri TK, Blum SI, et al Patient-reported outcomes of patients with advanced renal cell carcinoma (aRCC) treated with first-line nivolumab plus cabozantinib versus sunitinib: the CheckMate 9ER trial. J Clin Oncol. 2021;39(suppl 6; abstr 285).

[31] FDA approves nivolumab plus cabozantinib for advanced renal cell carcinoma. https://www.fda.gov/drugs/resources-information-approved-drugs/fda-approves-nivolumab-plus-cabozantinib-advanced-renal-cell-carcinoma. Accessed November 2021.

[32] Choueiri TK , Powles T, Burotto M, et al. Nivolumab plus Cabozantinib versus Sunitinib for Advanced Renal-Cell Carcinoma. N Engl J Med. 2021;384(9):829-841. 33657295 10.1056/NEJMoa2026982 PMC8436591

[33] Wang C , Thudium KB, Han M, et al In vitro characterization of the anti-PD-1 antibody nivolumab, BMS-936558, and in vivo toxicology in non-human primates. Cancer Immunol Res. 2014;2(9):846-856. 24872026 10.1158/2326-6066.CIR-14-0040

[34] Ribas A , Wolchok JD. Cancer immunotherapy using checkpoint blockade. Science. 2018;359(6382):1350-1355. 29567705 10.1126/science.aar4060 PMC7391259

[35] Apolo AB , Nadal R, Girardi DM, et al Phase I study of cabozantinib and nivolumab alone or with ipilimumab for advanced or metastatic urothelial carcinoma and other genitourinary tumors. J Clin Oncol. 2020;38(31):3672-3684. 32915679 10.1200/JCO.20.01652 PMC7605393

[36] Apolo AB , Nadal R, Tomita Y, et al Cabozantinib in patients with platinum-refractory metastatic urothelial carcinoma: an open-label, single-centre, phase 2 trial. Lancet Oncol. 2020;21(8):1099-1109. 32645282 10.1016/S1470-2045(20)30202-3 PMC8236112

[37] Motzer R , Alekseev B, Rha SY, et al; CLEAR Trial Investigators. Lenvatinib plus pembrolizumab or everolimus for advanced renal cell carcinoma. N Engl J Med. 2021;384(14):1289-1300. 33616314 10.1056/NEJMoa2035716

[38] Grünwald V , Powles T, Choueiri TK, et al Lenvatinib plus everolimus or pembrolizumab versus sunitinib in advanced renal cell carcinoma: study design and rationale. Future Oncol. 2019;15(9):929-941. 30689402 10.2217/fon-2018-0745

[39] Choueiri TK , Powles T, Burotto M, et al; CheckMate 9ER Investigators. Nivolumab plus cabozantinib versus sunitinib for advanced renal-cell carcinoma. N Engl J Med. 2021;384(9):829-841. 33657295 10.1056/NEJMoa2026982 PMC8436591

[40] Powles T , Plimack ER, Soulières D, et al Pembrolizumab plus axitinib versus sunitinib monotherapy as first-line treatment of advanced renal cell carcinoma (KEYNOTE-426): extended follow-up from a randomised, open-label, phase 3 trial. Lancet Oncol. 2020;21(12):1563-1573. 33284113 10.1016/S1470-2045(20)30436-8

[41] Zhang T , Ballman KV, Choudhury AD, et al PDIGREE: an adaptive phase III trial of PD-inhibitor nivolumab and ipilimumab (IPI-NIVO) with VEGF TKI cabozantinib (CABO) in metastatic untreated renal cell cancer (Alliance A031704). J Clin Oncol. 2020;38(suppl 15; abstr TPS5100).

[42] Choueiri TK , Albiges L, Powles T, et al A phase III study (COSMIC-313) of cabozantinib (C) in combination with nivolumab (N) and ipilimumab (I) in patients (pts) with previously untreated advanced renal cell carcinoma (aRCC) of intermediate or poor risk. J Clin Oncol. 2020;38(suppl 6; abstr TPS767).

[43] Diab A , Tannir NM, Bentebibel SE, et al Bempegaldesleukin (NKTR-214) plus nivolumab in patients with advanced solid tumors: phase I dose-escalation study of safety, efficacy, and immune activation (PIVOT-02). Cancer Discov. 2020;10(8):1158-1173. 32439653 10.1158/2159-8290.CD-19-1510

[44] Tannir NM , Agarwal N, Pal SK, et al PIVOT-09: a phase III randomized open-label study of bempegaldesleukin (NKTR-214) plus nivolumab versus sunitinib or cabozantinib (investigator’s choice) in patients with previously untreated advanced renal cell carcinoma (RCC). J Clin Oncol. 2020;38(suppl 6; abstr TPS763). 10.1200/JCO.23.02082 38838287

[45] Choueiri TK , Escudier B, Powles T, et al; METEOR Investigators. Cabozantinib versus everolimus in advanced renal cell carcinoma (METEOR): final results from a randomised, open-label, phase 3 trial. Lancet Oncol. 2016;17(7):917-927. 27279544 10.1016/S1470-2045(16)30107-3

[46] Choueiri TK , Escudier B, Powles T, et al; METEOR Investigators. Cabozantinib versus everolimus in advanced renal-cell carcinoma. N Engl J Med. 2015;373(19):1814-1823. 26406150 10.1056/NEJMoa1510016 PMC5024539

[47] Motzer RJ , Hutson TE, Glen H, et al Lenvatinib, everolimus, and the combination in patients with metastatic renal cell carcinoma: a randomised, phase 2, open-label, multicentre trial. Lancet Oncol. 2015;16(15):1473-1482. 26482279 10.1016/S1470-2045(15)00290-9

[48] Rini BI , Pal SK, Escudier BJ, et al Tivozanib versus sorafenib in patients with advanced renal cell carcinoma (TIVO-3): a phase 3, multicentre, randomised, controlled, open-label study. Lancet Oncol. 2020;21(1):95-104. 31810797 10.1016/S1470-2045(19)30735-1

[49] Pal SK , Escudier B, Atkins MB, et al TIVO-3: final OS analysis of a phase III, randomized, controlled, multicenter, open-label study to compare tivozanib to sorafenib in subjects with metastatic renal cell carcinoma (RCC). J Clin Oncol. 2020;38(Suppl 15; abstr 5062).

[50] U.S. Food and Drug Administration. *FDA Approves Tivozanib for Relapsed or Refractory Advanced Renal Cell Carcinoma*. https://www.fda.gov/drugs/resources-information-approved-drugs/fda-approves-tivozanib-relapsed-or-refractory-advanced-renal-cell-carcinoma. Accessed November 2021.

[51] Pal SK , Albiges L, Suarez Rodriguez C, et al CONTACT-03: randomized, open-label phase III study of atezolizumab plus cabozantinib versus cabozantinib monotherapy following progression on/after immune checkpoint inhibitor (ICI) treatment in patients with advanced/metastatic renal cell carcinoma. J Clin Oncol. 2021;39(suppl 6; abstr TPS370).

[52] AVEO oncology announces collaboration with Bristol Myers Squibb to evaluate FOTIVDA^®^ (tivozanib) in combination with OPDIVO^®^ (nivolumab) in pivotal phase 3 TiNivo-2 trial in IO relapsed renal cell carcinoma. Accessed March 12, 2021. https://bit.ly/3ldIasp.

[53] Courtney KD , Infante JR, Lam ET, et al Phase I dose-escalation trial of PT2385, a first-in-class hypoxia-inducible factor-2α antagonist in patients with previously treated advanced clear cell renal cell carcinoma. J Clin Oncol. 2018;36(9):867-874. 29257710 10.1200/JCO.2017.74.2627 PMC5946714

[54] Courtney KD , Ma Y, Diaz de Leon A, et al HIF-2 complex dissociation, target inhibition, and acquired resistance with PT2385, a first-in-class hif-2 inhibitor, in patients with clear cell renal cell carcinoma. Clin Cancer Res. 2020;26(4):793-803. 31727677 10.1158/1078-0432.CCR-19-1459 PMC7024660

[55] Papadopoulos KP , Jonasch E, Zojwalla NJ, Wang K, Bauer TM. A first-in-human phase 1 dose-escalation trial of the oral HIF-2a inhibitor PT2977 in patients with advanced solid tumors. J Clin Oncol. 2018;36(suppl 15; abstr 2508).

[56] Choueiri TK , Plimack ER, Bauer TM, et al Phase I/II study of the oral HIF-2 α inhibitor MK-6482 in patients with advanced clear cell renal cell carcinoma (RCC). J Clin Oncol. 2020;38(suppl 6; abstr 611).

[57] Toth AT , Cho DC. Emerging therapies for advanced clear cell renal cell carcinoma. J Kidney Cancer VHL. 2020;7(4):17-26. 33364146 10.15586/jkcvhl.2020.156 PMC7738296

[58] Choueiri TK , Albiges L, Fan L, et al Phase III study of the hypoxia-inducible factor 2α (HIF-2α) inhibitor MK-6482 versus everolimus in previously treated patients with advanced clear cell renal cell carcinoma (ccRCC). J Clin Oncol. 2020;38(suppl 15; abstr TPS5094).

[59] Choueiri TK , Bauer TM, McDermott DF, et al Phase 2 study of the oral hypoxia-inducible factor 2α (HIF-2α) inhibitor MK-6482 in combination with cabozantinib in patients with advanced clear cell renal cell carcinoma (ccRCC). J Clin Oncol. 2021;39(suppl 6; abstr 272).

[60] Yong C , Stewart GD, Frezza C. Oncometabolites in renal cancer. Nat Rev Nephrol. 2020;16(3):156-172. 31636445 10.1038/s41581-019-0210-z PMC7030949

[61] Tannir NM , Fan AC, Lee RJ, et al Phase 1 study of glutaminase (GLS) inhibitor CB-839 combined with either everolimus (E) or cabozantinib (Cabo) in patients (pts) with clear cell (cc) and papillary (pap) metastatic renal cell cancer (mRCC). J Clin Oncol. 2018;36(suppl 6):603.

[62] Tannir NM , Motzer RJ, Agarwal N, et al CANTATA: A randomized phase 2 study of CB-839 in combination with cabozantinib vs. placebo with cabozantinib in patients with advanced/metastatic renal cell carcinoma. J Clin Oncol. 2018;36(suppl 15):TPS4601.

[63] Meric-Bernstam F , Lee RJ, Carthon BC, et al CB-839, a glutaminase inhibitor, in combination with cabozantinib in patients with clear cell and papillary metastatic renal cell cancer (mRCC): results of a phase I study. J Clin Oncol. 2019;37(suppl 7; abstr 549).

[64] Motzer RJ , Lee C-H, Emamekhoo H, et al ENTRATA: Randomized, double-blind, phase II study of telaglenastat (tela; CB-839) + everolimus (E) vs placebo (pbo) + E in patients (pts) with advanced/metastatic renal cell carcinoma (mRCC). Ann Oncol. 2019;30:v889-v890.

[65] Tannir NM , Agarwal N, Porta C, et al CANTATA: primary analysis of a global, randomized, placebo (Pbo)-controlled, double-blind trial of telaglenastat (CB-839) + cabozantinib versus Pbo + cabozantinib in advanced/metastatic renal cell carcinoma (mRCC) patients (pts) who progressed on immune checkpoint inhibitor (ICI) or anti-angiogenic therapies. J Clin Oncol. 2021;39(suppl 15; abstr 4501).

[66] Bex A , Mulders P, Jewett M, et al Comparison of immediate vs deferred cytoreductive nephrectomy in patients with synchronous metastatic renal cell carcinoma receiving sunitinib: the SURTIME randomized clinical trial. JAMA Oncol. 2019;5(2):164-170. 30543350 10.1001/jamaoncol.2018.5543 PMC6439568

[67] Mejean A , Thezenas S, Chevreau C, et al Cytoreductive nephrectomy (CN) in metastatic renal cancer (mRCC): update on Carmena trial with focus on intermediate IMDC-risk population. J Clin Oncol. 2019;37(suppl 15; abstr 4508).

[68] Bakouny Z , Xie W, Dudani S, et al Cytoreductive nephrectomy (CN) for metastatic renal cell carcinoma (mRCC) treated with immune checkpoint inhibitors (ICI) or targeted therapy (TT): a propensity score-based analysis. J Clin Oncol. 2020;38(suppl 6; abstr 608).

[69] Flacco ME , Manzoli L, Ioannidis JP. Noninferiority is almost certain with lenient noninferiority margins. J Clin Epidemiol. 2016;71:118. 10.1016/j.jclinepi.2015.11.010 26607237

[70] Soonawala D , Dekkers OM, Vandenbroucke JP, Egger M. Noninferiority is (too) common in noninferiority trials. J Clin Epidemiol. 2016;71:118-120. 10.1016/j.jclinepi.2015.11.009 26607238

[71] Lalani A-KA , Swaminath A, Pond GR, et al Phase II trial of cytoreductive stereotactic hypofractionated radiotherapy with combination ipilimumab/nivolumab for metastatic kidney cancer (CYTOSHRINK). J Clin Oncol. 2020;38(suppl 6; abstr TPS761).

[72] Choueiri TK , Atkins MB, Bakouny Z, et al Summary from the first kidney cancer research summit, September 12-13, 2019: a focus on translational research. J Natl Cancer Inst. 2021;113(3):234-243. 32359162 10.1093/jnci/djaa064 PMC7936057

